# A new machine learning model for predicting severity prognosis in patients with pulmonary embolism: Study protocol from Wenzhou, China

**DOI:** 10.3389/fninf.2022.1052868

**Published:** 2022-12-16

**Authors:** Hang Su, Yeqi Shou, Yujie Fu, Dong Zhao, Ali Asghar Heidari, Zhengyuan Han, Peiliang Wu, Huiling Chen, Yanfan Chen

**Affiliations:** ^1^College of Computer Science and Technology, Changchun Normal University, Changchun, Jilin, China; ^2^Department of Pulmonary and Critical Care Medicine, The First Affiliated Hospital of Wenzhou Medical University, Wenzhou, China; ^3^School of Surveying and Geospatial Engineering, College of Engineering, University of Tehran, Tehran, Iran; ^4^College of Computer Science and Artificial Intelligence, Wenzhou University, Wenzhou, Zhejiang, China

**Keywords:** feature selection, extreme learning machine, disease diagnosis, swarm intelligence, pulmonary embolism

## Abstract

**Introduction:**

Pulmonary embolism (PE) is a common thrombotic disease and potentially deadly cardiovascular disorder. The ratio of clinical misdiagnosis and missed diagnosis of PE is very large because patients with PE are asymptomatic or non-specific.

**Methods:**

Using the clinical data from the First Affiliated Hospital of Wenzhou Medical University (Wenzhou, China), we proposed a swarm intelligence algorithm-based kernel extreme learning machine model (SSACS-KELM) to recognize and discriminate the severity of the PE by patient’s basic information and serum biomarkers. First, an enhanced method (SSACS) is presented by combining the salp swarm algorithm (SSA) with the cuckoo search (CS). Then, the SSACS algorithm is introduced into the KELM classifier to propose the SSACS-KELM model to improve the accuracy and stability of the traditional classifier.

**Results:**

In the experiments, the benchmark optimization performance of SSACS is confirmed by comparing SSACS with five original classical methods and five high-performance improved algorithms through benchmark function experiments. Then, the overall adaptability and accuracy of the SSACS-KELM model are tested using eight public data sets. Further, to highlight the superiority of SSACS-KELM on PE datasets, this paper conducts comparison experiments with other classical classifiers, swarm intelligence algorithms, and feature selection approaches.

**Discussion:**

The experimental results show that high D-dimer concentration, hypoalbuminemia, and other indicators are important for the diagnosis of PE. The classification results showed that the accuracy of the prediction model was 99.33%. It is expected to be a new and accurate method to distinguish the severity of PE.

## 1 Introduction

Venous thromboembolism (VTE) is a general term for deep vein thrombosis (DVT) and pulmonary embolism (PE). They are fundamentally different manifestations of a disease, but the difference lies in their different parts and stages (Desai et al., 2021). Behind myocardial infarction and stroke, VTE is the third most usual type of cardiovascular disease (Duffett et al., 2020; Yang et al., 2021). PE is the general name of a company of diseases or clinical syndromes aroused by various embolus embolizing pulmonary artery or its branches, including pulmonary thromboembolism (PTE), fat embolism syndrome, amniotic fluid embolism, air embolism and so on (Yang et al., 2021). In the European Union, PE is diagnosed in 1–2 out of 1000 people annually (Cohen et al., 2007). The study found that the incidence of PE was correlated with age, race, and gender (Heit, 2015). Any factors leading to venous stasis, tissue trauma or hypercoagulable state (Virchow’s triad) are a risk for PE (Lurie et al., 2019).

Pulmonary embolism is a fatal disease, and the mortality rate has been up to 30% in studies including autopsy-based PE diagnosis (Cushman, 2007). Risk stratification and early intervention for acute pulmonary embolism (APE) are beneficial in reducing mortality. Patients with hemodynamic instability are considered to be at high risk for early death (Konstantinides et al., 2020). However, for patients with stable hemodynamics, it is necessary to carry out early risk assessment employing clinical symptoms, laboratory indicators, and imaging. Pruszczyk et al. (2021) developed an early risk assessment scale for hemodynamically stable PE. Another study found that computed tomographic pulmonary angiography (CTPA) was helpful for risk stratification (Gao et al., 2021). Although many risk-assessment methods, such as the Wells score and revised Geneva score (Klok et al., 2008b; van Es et al., 2017; Glober et al., 2018; Triantafyllou et al., 2021), have been proposed, stratification in the intermediate-high-risk populations remains challenging and remains an important area of research we need to conduct. Nowadays, a new machine learning method is used to analyze the effect of biomarkers on risk stratification of PE.

Numerous diagnostic tools are available to help clinicians achieve effective conclusions (Li et al., 2022; Liu S. et al., 2022; Liu Z. et al., 2022). There are different claims regarding the performance advantages and disadvantages of machine learning algorithms and traditional statistical methods for diagnosis and prediction. Statistical methods are relatively mature, relying on their simplicity and flexibility to first filter out relevant indicators and then construct multivariate logistic regression or linear regression models, etc., for aiding the diagnosis of EMs. However, machine learning, a novel scientific approach that integrates the benefits of statistics and other disciplines, has also proven its capability to handle induction and analysis of big data, non-linear and complex problems. Then, the following is the current state of research on machine learning techniques for complementary medical diagnosis.

Too and Mirjalili (2021) introduced the equilibrium optimization algorithm (EO) into the feature selection method and proposed a wraparound feature selection model for finding important features of biological data. Lambert and Perumal (2021) proposed a high-performance classifier model for the diagnosis of chronic kidney disease using a dyadic-based Firefly optimization algorithm combined with deep neural networks. Kitonyi and Segera (2021) proposed an improved classification prediction model based on the gray wolf algorithm and gradient descent algorithm for improving the performance of feature selection algorithms for medical data processing, and the model has high accuracy and stability. Hu and Razmjooy (2021) proposed a deep neural network model on the basis of a metaheuristic technique for segmentation, feature extraction, and classification of brain tumors, and the experimental results showed that this model is excellent in automaticity. Canayaz (2021) introduced the particle swarm optimization algorithm (PSO) and gray wolf optimization algorithm into the support vector machine (SVM) for preprocessing of COVID-19 images, and the total accuracy of the suggested model was 99.38%. Mazaheri and Khodadadi (2020) used a metaheuristic algorithm to remove redundant features and then combined it with a machine learning algorithm to propose a medical aid system for heart disease classification with the highest accuracy of 98.75%. Vijayashree and Sultana (2018) put forward a classification model combining the PSO with SVM on the basis of population diversity function and tuning function, which can effectively classify vast amounts of medical data. Medjahed et al. (2017) presented a cancer diagnosis model according to kernel learning and feature selection, which can augment the classification accuracy of the model using the reduced number of genes. It can be seen that more and more researchers are using machine learning techniques to diagnose diseases and pay much attention to the classification accuracy and classification efficiency of classifiers. Since medical images are characterized by a large amount of input information, different sources, and high complexity, the ability of diagnostic models to handle large data and high-dimensional data relationships becomes critical. Therefore, researchers have started to use non-gradient descent metaheuristic algorithms for optimizing the diagnostic accuracy and diagnostic efficiency of the models.

Most conventional optimization algorithms must to interact with information pertaining to the showcase space’s exterior or require an asynchronous method to handle problems (Zhang et al., 2021). Meta-heuristic algorithms, also known as swarm intelligence algorithms (SIAs), are often utilized to mine optimal or satisfactory solutions to complex optimization situations. As a mechanism based on computational intelligence, these algorithms are robust, self-organizing and flexible. Compared with traditional optimization methods (Newton’s, simplex, enumeration, etc.), they have a greater improvement in problem-solving time, scientific layout, and rational allocation of resources and are widely used in signal processing, image processing, production scheduling, and mechanical design. Over the last few years, more and more SIAs have been put forward, such as differential evolution (DE) (Storn and Price, 1997), sine cosine algorithm (SCA) (Mirjalili, 2016), salp swarm algorithm (SSA) (Mirjalili et al., 2017), whale optimizer (WOA) (Mirjalili and Lewis, 2016), moth-flame optimization (MFO) (Mirjalili, 2015), particle swarm optimization (PSO) (Kennedy and Eberhart, 1995), fruit fly optimization algorithm (FOA) (Pan, 2012), chaotic BA (CBA) (Adarsh et al., 2016), improved ant colony optimizer (RCACO) (Zhao et al., 2020), chaotic SCA (Ji et al., 2020), moth-flame optimizer with sine cosine mechanisms (SMFO) (Chen et al., 2021), improved WOA (EWOA) (Tu et al., 2020), and so on. Cuckoo search (CS) (Yang and Suash, 2009) is a SIA proposed by British scholars Xin-She Yang and Suash Deb in 2009, motivated by the behavior of cuckoo’s parasitic egg production hatching in nature. It is broadly employed in various fields since its low complexity and excellent effectiveness in finding the best. Rosli and Mohamed (2021) presented a developed CS approach with different spaces for optimizing the Bouc-Wen model in magnetorheological damper applications. Mohiz et al. (2021) submitted an enhanced CS algorithm on the basis of a greedy strategy for optimizing task placement on a network-on-chip (NoC) core. Bibiks et al. (2018) proposed an improved discrete CS (IDCS) to solve the resource-constrained project scheduling problems (RPs). Li X. et al. (2017) raised an improved cuckoo search (ICS) method to optimize the disparity pattern of monopulse antennas. Lim et al. (2016) combined the benefits of genetic algorithm into the CS algorithm to propose a new hybrid algorithm for optimizing hole-making operations in engineering. Long et al. (2014) proposed a hybrid cuckoo pattern search algorithm (HCPS) according to feasibility rules for handling constrained numerical and engineering design problems. As can be seen, the CS algorithm is widely used in optimization problems in various fields and is a high-performance optimization algorithm.

According to Macerday’s No Free Lunch theory, no single optimization method can be applied to every optimization problem, and the CS algorithm is no exception. The characteristics of the Lévy flight formulation lead to large randomness in the CS algorithm’s search for the best solution, which allows the algorithm to compare most solutions during the search phase and facilitates the fast identification of the better solution. However, due to its high randomness, the algorithm is often unable to find a better solution in the later exploitation stage, thus dropping into the local optimum (LO) trap and wasting the performance of the later iterations of the algorithm. Therefore, to solve the dilemma of CS algorithm in the later period, an improved version of the SSACS algorithm is put forward by introducing the SSA.

For the sake of proving the overall performance of the SSACS algorithm, the classical CEC2014 benchmark function test set is used to test the algorithm comprehensively. During the benchmark function testing, SSACS is compared with five well-known basic algorithms and five latest advanced ones to demonstrate the superiority of the algorithm. Further, to augment the accuracy in the feature selection process of the PE dataset, an improved version of the KELM classification prediction model (SSACS-KELM) based on SSACS is suggested. To thoroughly test the adaptability and stability of SSACS-KELM, eight datasets of different sizes are used in this paper. To further demonstrate that the combination of bSSACS and KELM is excellent, this experiment combines bSSACS with four different classifiers. Then, to show the difference in performance between bSSACS-KELM and classical methods, this experiment compares bSSACS with five classical methods. Furthermore, to illustrate the advantages of bSSACS over other algorithms of the same type, this experiment is designed to compare bSSACS with nine other swarm intelligence algorithms. For the purpose of more accurately assessing the processing capability of the bSSACS-KELM model on PE data, the experiments in this paper will use four metrics, such as accuracy, precision, specificity, and Matthews correlation coefficient (MCC), to perfectly emphasis the reliability of the classification outcomes. Finally, the results of the experiments, after 10-fold cross-validation, yield the five most critical features of the PE dataset.

The main contributions of this paper are as below: (1) In this paper, a pulmonary embolism-assisted diagnosis model with higher performance is presented, which can accurately select the effective features of the PE set and provide valuable diagnostic information for physicians. (2) This paper combines the improved swarm intelligence algorithm with the KELM classifier to propose a classifier with higher accuracy, which provides a high-performance classifier for the feature selection situation. (3) In this paper, a swarm intelligence algorithm (SSACS) with a more robust exploration capability and higher convergence accuracy is proposed and validated against peers.

The remainder of this paper is structured as below. Section “2 Data and methods” describes the PE dataset, the CS algorithm, the SSA, and the KELM classifier at length. In Section “3 The proposed method,” the SSACS algorithm and the SSACS-KELM model are put forward. In Section “4 Results and discussion,” we validate and analyze the performance of the SSACS algorithm and the SSACS-KELM model. In Section “5 Discussion,” the paper combines practical medical knowledge and experimental results for a detailed discussion. Eventually, Section “6 Conclusion and future works” sums up the whole content and guides the future work.

## 2 Data and methods

In this section, the source of the PE dataset and its acquisition criteria are first described. Then, an overview of the original CS algorithm, SSA algorithm, and KELM classifier is presented.

### 2.1 PE data collection

The Ethics Committee of the Affiliated of the Wenzhou Medical University agreed on the present study (ethical approval code: KY2021-R097). Our study selected patients diagnosed with pulmonary embolism at the First Affiliated Hospital of Wenzhou Medical University from April 2014 to May 2020. For this retrospective study, PE was confirmed by CTPA, echocardiography (ECHO), or the value of ventilation-perfusion (V/Q) scintigraphy in patients with symptoms suggestive of PE (Konstantinides et al., 2020). Besides, we excluded patients who have taken anticoagulant or antiplatelet drugs recently.

According to the European Society of Cardiology (ESC) guidelines for diagnosing and managing acute PE in 2014 (Konstantinides et al., 2014), patients with the following manifestations are defined as high risk: (I) shock; (II) Hypotension: systolic blood pressure <90 mmHg or pressure drop >40 mmHg for more than 15 min, excluding new arrhythmia, hypovolemia, or hypotension due to sepsis. A total of 142 patients we screened were divided according to the guidelines into two groups: intermediate-low-risk PE (*n* = 73) and high-risk PE (*n* = 69). For each PE patient, information on the general data (gender, age, vital signs, and past medical history) and serum biomarkers were collected. We also recorded whether patients had dyspnea, chest pain, hemoptysis, or syncope during the onset of the disease.

The data were analyzed and processed by the statistical software SPSS 21.0 and were stated as the mean ± standard deviation ($\bar{\chi }$ ± SD). All continuous variables met the normality, and the independent sample *t*-test procedure was employed to analyze continuous variables. The Chi-square test was used for categorical variables. A *p*-value < 0.05 stood for statistically significant. All sick people’s general data and serum biomarkers were described in Table 1. The results from chi-square tests and independent sample *t*-test are presented in Tables 2, 3, respectively.

**TABLE 1:** A complete list of the features used in this study and their definitions number.

	Features	Abbreviation
F1	Age	Age
F2	Gender	Gender
F3	Dyspnea	Dyspnea
F4	Chest pain	CP
F5	Hemoptysis	Hemoptysis
F6	Syncope	Syncope
F7	Cardiopulmonary resuscitation	CPR
F8	Altered mental status	AMS
F9	Chronic heart failure	CHF
F10	Chronic lung disease	CLD
F11	History of tumor	HOT
F12	Systolic blood pressure (mmHg)	SBP
F13	Diastolic blood pressure (mmHg)	DBP
F14	Pulse rate (times/min)	PR
F15	Temperature (°C)	T
F16	Respiratory rate (times/min)	RR
F17	Troponin I (μg/L)	cTnI
F18	N-terminal-pro basic natriuretic peptide (pg/ml)	NT-proBNP
F19	D-dimer (mg/L)	D-D
F20	Alanine aminotransferase (U/L)	ALT
F21	Albumin (g/L)	ALB
F22	Total bilirubin (μmol/L)	TBIL
F23	Direct bilirubin (umol/L)	DBIL
F24	Creatinine (μmol/L)	Cr
F25	Glucose concentration (mmol/L)	Glu
F26	Potassium ion concentration (mmol/L)	K^+^
F27	Sodium ion concentration (mmol/L)	Na^+^
F28	Chloride ion concentration (mmol/L)	Cl^–^
F29	Calcium ion concentration (mmol/L)	Ca^2+^

**TABLE 2:** Clinical characteristics in intermediate-low-risk PE patients and high-risk PE patients.

Index	Intermediate-low-risk PE (*n* = 73)	High-risk PE (*n* = 69)	χ^2^ value	*P*-value
Gender (male/female)	43/30	30/39	3.379	0.066
Dyspnea (no/yes)	32/41	26/43	0.556	0.456
CP (no/yes)	59/14	61/8	1.558	0.213
Hemoptysis (no/yes)	69/4	67/2	0.120	0.729
Syncope (no/yes)	70/3	49/20	16.171	0.000
CPR (no/yes)	73/0	61/8	6.921	0.009
AMS (no/yes)	69/4	55/14	7.029	0.008
CHF (no/yes)	58/15	55/14	0.001	0.969
CLD (no/yes)	58/15	61/8	2.095	0.148
HOT (no/yes)	62/11	47/22	2.095	0.018

**TABLE 3:** General data and serum biomarkers in intermediate-low-risk PE patients and high-risk PE patients.

Index	Intermediate-low-risk PE (*n* = 73)	High-risk PE (*n* = 69)	*P*-value
Age	65.49 ± 13.313	64.65 ± 11.732	0.691
SBP	118.26 ± 21.321	86.90 ± 8.168	0.000
DBP	70.37 ± 11.881	54.51 ± 9.646	0.000
PR	88.82 ± 14.904	92.13 ± 25.532	0.351
T	37.15 ± 0.579	37.20 ± 1.291	0.749
RR	20.11 ± 2.525	21.09 ± 5.412	0.175
cTnI	0.39 ± 0.542	2.91 ± 20.066	0.300
NT-proBNP	324.59 ± 389.660	446.62 ± 620.749	0.166
D-D	5.09 ± 5.330	9.11 ± 8.215	0.001
ALT	49.34 ± 99.048	54.83 ± 79.260	0.717
ALB	34.037 ± 4.600	32.47 ± 4.472	0.042
TBIL	11.99 ± 6.931	12.48 ± 8.702	0.710
DBIL	5.06 ± 4.166	5.46 ± 4.877	0.594
Cr	69.79 ± 24.289	85.62 ± 77.273	0.108
Glu	6.39 ± 2.188	8.12 ± 5.745	0.018
K^+^	3.79 ± 0.437	3.91 ± 0.502	0.130
Na^+^	139.68 ± 3.472	138.10 ± 3.647	0.009
Cl^–^	103.36 ± 4.204	102.81 ± 5.857	0.524
Ca^2+^	2.18 ± 0.137	2.15 ± 0.147	0.136

### 2.2 Mathematical model of CS algorithm

The CS algorithm is an intelligent bionic method suggested by Yang and Suash (2009). The algorithm has attracted a lot of attention from scholars at home and abroad because of its advantages of few parameters, easy implementation, robustness, and success in solving practical problems such as function optimization and engineering optimization.

The CS algorithm performs a random search of the target space by simulating the parasitic brood-rearing behavior of cuckoo species. The algorithm chiefly comes from the following three assumptions: (i) every cuckoo lays one egg at a time and chooses its nest arbitrarily; (ii) the eggs deposited in the best nest can hatch and generate a new generation; (iii) the number of nests selected for egg-laying is finite and is found by the nest owner with probability *P*_*a*_ ∈ [0,1] after the cuckoo egg is thrown out of the nest or the nest owner abandons the nest and rebuilds a new nest in another place. The fundamental flow of the algorithm is.

(1) Randomly produce *L* nest locations in the solution space (i.e., corresponding to *L* solutions), calculate the fitness of each nest using the set fitness function, and keep the best location, and iterate over the rest.
(2) Assume that the current number of selected generations is *k* nests numbered *i* for the location of $x_{i}^{4}=\left(x_{i,1},x_{i,2},\cdots ,x_{i,D}\right)$, where 1⩽i⩽L, *D* is the number of dimensions of the problem solved, in addition to the optimal direct retention to the next generation, the rest of further selected generations, that is

(1) $$x\left(k+1\right)=x\left(k\right)+a\times k^{-\lambda },\left(1<\lambda ⩽3\right)$$

where, *a* > 0, is the step control quantity, whose size is mainly determined by the scale of the problem-solving; *k*^−λ^ is the random distribution function obeying Lévy’s law.

(3) Assume that the probability of the nest owner finding cuckoo eggs is *P_a_*, and randomly generate positive numbers *r* ∈ [0,1] obeying uniform distribution, if *r* > *P*_*a*_., then the cuckoo eggs are thrown out of the nest or the nest is abandoned to regenerate a new nest; otherwise, it remains unchanged.
(4) Decide whether the set number of selected generations is satisfied; otherwise, go back to step (2) and go on to select and update the generations until the selection conditions are met.

The pseudo-code of the CS algorithm is displayed in Algorithm 1.

**Algorithm 1:** Pseudo-code of CS.

Initialize fitness function *f*(*x*),*x* = (*x*_1_,*x*_2_,*x*_3_,⋯,*x*_*d*_)*^T^* Initialize the number of iterations *t* = 0, discovered parameter *P*_*a*_ = 0.25, and population size *N* Initialize the initial value of each individual $x_{i}^{0}\left(i=1,2\ldots ,N\right)$ in the population **While** *l* ≤ *Maximum number of iterations* Use Lévy flight Calculate the fitness $f_{new,i}^{t}$ for the updated agent $x_{new,i}^{t}$ Randomly choose a candidate individual $x_{j}^{t}$ from the population **If** the fitness of the updated agent is better Replace the new agent $x_{new,i}^{t}$ for the candidate agent $x_{j}^{t}$ **End If** Replace some of the suboptimal solutions with randomly generated ones with *P_a_* probability The next generation keeps better solutions Find and maintain optimum population solutions Update iteration numbers *t* = *t* + 1 **End While** Return the best solution

### 2.3 Mathematical model of the SSA algorithm

The salp swarm algorithm (SSA) (Mirjalili et al., 2017) was proposed in 2017, which achieves the exploration of solution space by simulating the foraging behavior of the Bottlenose Sea Squirt in the ocean in nature. The method has been successfully applied to deal with the situations of photovoltaic system optimization, feature extraction, image processing, and biomedical signal processing with its remarkable features of fast convergence, robustness, and easy implementation.

Salp swarm algorithm searches for the optimal solution to the problem by simulating the behavior of the group chain movement of the bottle sea squirt in the ocean, the algorithm separates the individuals into leaders and followers, and these two types of individuals take different movement updates: leaders are located at the front of the group chain and guide the population movement according to the position of the elite individuals; followers are located at the back of the group chain and follow each other’s movement. The process of SSA implementation is as follows.

(1) Set the maximum number of selected generations *t*_*max*_, initialize the number of selected generations *t* = 1, and establish the initial swarm {*X*_*i*_}(i=1, 2,…,N) with the number of individuals *N* and dimension *D*, where $X_{i}=\left(x_{i}^{1},x_{i}^{2},\cdots ,x_{i}^{D}\right)$.
(2) Calculate all individual fitness values, rank agents in accordance with their fitness, and select the current best agent as the elite individual *G*(*t*).
(3) Selecting the top *N*/2 individuals of the population as leaders, i.e., i⩽N/2 updating the leader individual positions according to Eq. (2).

(2) $$X_{i}^{j}\,\left(t+1\right)=\left\{ \begin{array}{c} G_{j}\,\left(t\right)+c_{1}\,\left[c_{2}^{*}\,\,\left(u\,b_{j}-l\,b_{j}\right)+l\,b_{j}\right],c_{3}⩾0.5\\ G_{j}\,\left(t\right)-c_{1}\,\left[c_{2}^{*}\,\,\left(u\,b_{j}-l\,b_{j}\right)+l\,b_{j}\right],c_{3}<0.5 \end{array}\right.$$

where, *G*_*j*_(*t*) is the j-th dimensional component of the current elite individual, $X_{i}^{j}\,\left(t+1\right)$ is the j-th dimensional component of the leader individual i after the selection of generations; *ub*_*j*_,*lb*_*j*_ are the upper and lower bounds of the position of the j-th dimensional component of the bottle sheath individual: *c*_2_,*c*_3_, are the random numbers between [0,1]: *c*_*1*_ is the convergence coefficient, which gradually decreases with the increase of the selection of generations.

(4) Select *N*/2 individuals after the population as followers, i.e., *i* > *N*/2 update the position of the following individuals according to Eq. (3):


(3) Xij⁢(t+1)=0.5*⁢[Xij⁢(t)+Xi-1j⁢(t)]

where $X_{i}^{j}\,\left(t\right),X_{i-1}^{j}\,\left(t\right)$ denote the j-th dimensional component of the i-th and (i−1)-th population individuals after *t* selection generations, respectively, and $X_{i}^{j}\,\left(t+1\right)$ represents the j-th dimensional component of the updated population following agent *i*.

(5) Update the number of selection generations *t*, judge whether the maximum number of selection generations is satisfied, if not reach jump to step (2), otherwise output the elite individual position *G*(*t*), which is the global optimal solution.

The pseudo-code of the SSA is shown in Algorithm 2.

**Algorithm 2:** Pseudo-code of SSA.

Initialize parameters, population size *N*, maximum number of iterations *l* Initialize target function *f*(*x*),*x* = (*x*_1_,*x*_2_,*x*_3_,⋯,*x*_*d*_)*^T^* **While** *l* ≤ *Maximum number of iterations* **for** *i* = 1:*N* **if** *i* ≤ *N*/2 Update the leader position according to Eq. (2) **Else** Update the follower position according to Eq. (3) **End If** **End for** Transboundary treatment of bottled sea squirt individuals beyond the boundary Find and maintain optimum population solutions **End While** Return the best solution

### 2.4 KELM

The ELM model is a single hidden layer feedforward network presented by Glober et al. (2018) based on the generalized inverse matrix theory. Since the connection weights of the input layer to the implicit layer and the bias of the implicit layer of the ELM model do not need to be set artificially, it has the benefits of simple structure, fast operation, and good generalization performance, so it has been widely used in classification and regression problems. When given a training sample *S* = {(*x*_*n*_,*y*_*n*_),*n* = 1,2⋯,*N*}, the model representation is shown in Eq. (4).


(4) $$\hat{y}=g\,\left(\omega \,x+b\right)\,\beta =h\,\left(x\right)\,\beta$$

where: ω is the weight of the input and implied layers; *b* is the implied layer bias.

*g*(⋅) is the activation function; and β is the output weight.

The linear equation system *Y* = *H*β is solved by the least squares method, and the regularization factor *C* is introduced to enhance the generalization performance of the ELM model, and the output weight β expression is given in Eq. (5).


(5) $$\beta =H^{T}\,{\left(H\,H^{T}+\frac{I}{C}\right)}^{-1}\,Y$$

where: *I* is the diagonal matrix; *Y* is the desired output, based on which Glober et al. (2018) proposed the KELM model to replace the random mapping in the ELM model with the kernel mapping. Define the kernel matrix Ω = *HH^T^* and the matrix element Ω_*ELMi*,*j*_ = *h*(*x*_*i*_)*h*(*x*_*j*_) = *K*(*x*_*i*_,*x*_*j*_), where *K*(⋅) is the kernel function. At this point the output function of the KELM model can be stated as Eq. (6).


(6) $$\begin{array}{c} \hat{y}=h\,\left(x\right)\,\beta =h\,\left(x\right)\,H^{T}\,{\left(H\,H^{T}+\frac{I}{C}\right)}^{-1}\,Y=\\ {\left[\begin{array}{c} K\,\left(x,x_{1}\right)\\ \vdots \\ K\,\left(x,x_{N}\right) \end{array}\right]}^{T}\,{\left(\Omega_{E\,L\,M}+\frac{I}{C}\right)}^{-1}\,Y \end{array}$$

This paper selects the radial basis kernel function with strong localization and good generalization, and the expression obtained is shown in Eq. (7).


(7) $$K\left(x_{i},x_{j}\right)=\text{exp}\left(-\gamma ||x_{i},x_{j}|{|}^{2}\right)$$

## 3 The proposed method

In this section, this paper first introduces the core update method of SSA into the CS algorithm to propose the SSACS algorithm to boost the exploitation capability of the original CS and the possibility of getting rid of LO. Since the SSACS algorithm applies to continuous optimization problems, it is unsuitable for discrete feature selection situations. Consequently, this paper presents a discrete version of SSACS (bSSACS) based on S-type functions. Further, this paper combines the adapted bSSACS algorithm with the KELM classifier to propose a hybrid bSSACS-KELM model with stronger performance.

### 3.1 The proposed SSACS

**FIGURE 1:**
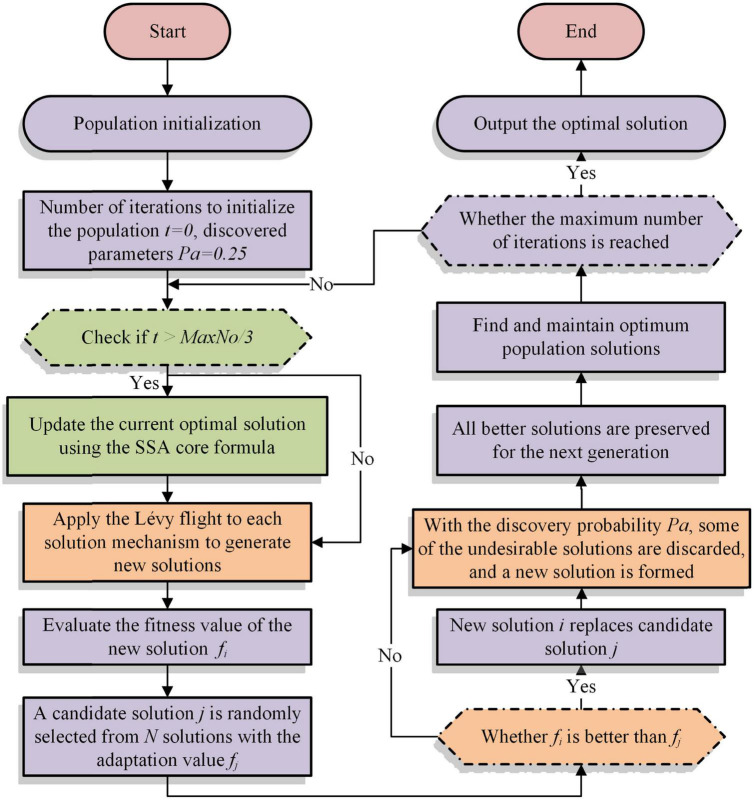
Flowchart of SSACS.

The core of the CS algorithm uses the Lévy flight function to update the optimal solution. The strong randomness of the Lévy flight function leads to the problem of solid search ability at the early phase and weak exploitation capability at the later phase during the iteration process. On the contrary, due to the characteristics of the SSA, the later exploitation step of the algorithm needs a large number of excellent samples to cross-borrow from each other to obtain a better solution. Therefore, to increase the efficiency of the search and better exploit the best solution in the later stage of the CS algorithm, the SSA with stronger exploitation capability is introduced in the second half of the iterative process of the algorithm. Among them, the pseudo-code and flowchart of SSACS are shown in Algorithm 3 and Figure 1, respectively.

**Algorithm 3:** Pseudo-code of SSACS.

Initialize the fitness function and the initial bird nest location *f*(*x*),*x* = (*x*_1_,*x*_2_,*x*_3_,⋯,*x*_*d*_)*^T^* Population size: *N* nests (*i* = 1,2,…,*N*) **While** *t* ≤ Maximum number of iterations (MaxNo) **If** *t* > *MaxNo*/3 Update the current optimal solution using the SSA core formula **End If** Using Lévy flights to create a new solution $x_{new,i}^{t}$ Calculate the fitness value $f_{new,i}^{t}$ for the updated agent $x_{new,i}^{t}$ Randomly choose a candidate individual $x_{j}^{t}$ from the population **If** the fitness of the updated agent is better Replace the new agent $x_{new,i}^{t}$ for the candidate agent $x_{j}^{t}$ **End If** Replace some of the suboptimal solutions with randomly generated ones with *P_a_* probability The next generation keeps better solutions Find and maintain optimum population solutions Update iteration numbers *t* = *t* + 1 **End While** Output the best solution

The complexity of SSACS mainly covers the introduced SSA, the fast sorting algorithm, and the fitness calculation. First, the complexity of SSA is *O*(*n*^2^). Then, the complexity of the quick sort algorithm in the best and worst case is *O*(n*logn)*andO*(*n*^2^), respectively. Finally, the complexity level of the fitness calculation is *O*(n*logn). As a result, the total complexity level of the SSACS algorithm is $O\;(SSACS)\;=\;O({\left(n+\log n\right)}^{*}\;n).$

### 3.2 The proposed feature selection model

#### 3.2.1 Discretization of SSACS

The SSACS algorithm based on the CS algorithm, like CS, generally solves continuous optimization problems. If the SSACS algorithm is applied to feature selection, it is necessary to discrete the SSACS algorithm. bSSACS algorithm converts CS into one that can handle discrete questions using a binary approach.

The bSSACS model transforms the original continuous space into binary one, where “1” means that the corresponding feature is selected to participate in the learning process, and “0” means the opposite. The algorithm first initializes the nest, then records the nest adaptation value by selecting generations, determines whether other eggs are found and Lévy’s flight to update the nest, and finally obtains the optimal solution after iteration. The individual’s initialization with binary representation uses a random threshold as Eq. (8).


(8) $$X_{d}\,\left(t+1\right)=\left\{ \begin{array}{cc} 1,\mathrm{sigmoid}\,\left(X_{d}\,\left(t\right)\right)\geq r\,\mathrm{and} & \\ \;0,\mathrm{otherwise} & \end{array}\right.$$

where *X*_*d*_(*t* + 1) is the solution of t-th next iteration, rand stands for a random number in the range of [0,1].

Sigmoid equation in this study is shown as Eq. (9).


(9) $$s\,i\,g\,m\,o\,i\,d\,\left(x\right)=\frac{1}{1+e^{-2\,x}}$$

where *x* denotes the solution produced by the proposed SSACS method.

#### 3.2.2 SSACS-KELM model

**FIGURE 2:**
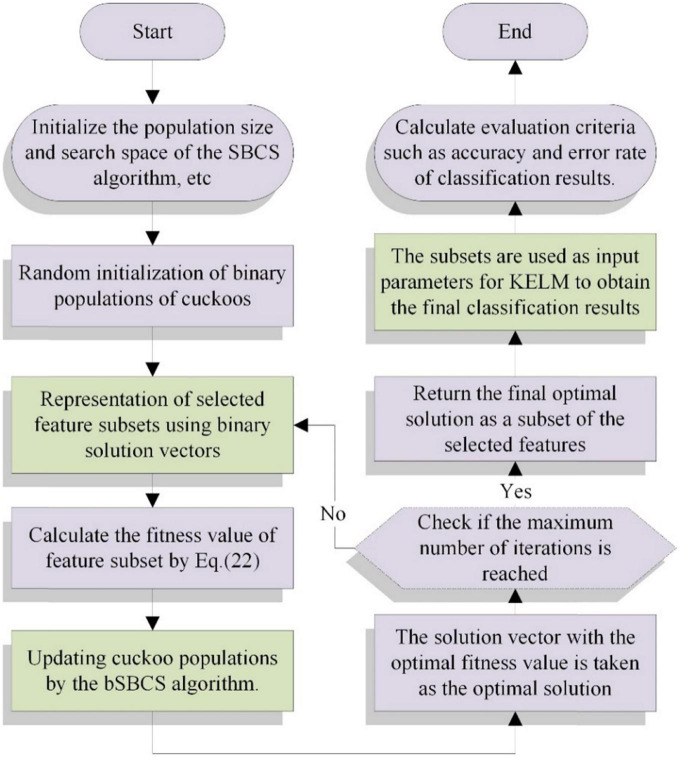
Flowchart of bSSACS-KELM.

In this section, SSACS algorithm based on KELM is proposed for feature selection. The discrete bSSACS algorithm is used to gain the optimal feature subset of PE dataset for feature selection. Then, the subsets are used as input parameters for KELM to obtain the final classification results. Basic framework of bSSACS-KELM is presented in Figure 2.

In particular, when bSSACS is combined with the KELM classifier, its corresponding fitness function is again too simple and not universal. When the SSACS algorithm is applied to feature selection, it is necessary to reconsider how to set the fitness function. There are two main requirements for feature selection as a data pre-processing process. First, the obtained feature subset has a high classification accuracy; that is, the feature subset requires to be acquired with strong relevance to the class; second, the obtained feature subset contains as few features as possible so as to prevent dimensional disasters. Therefore, in this paper, an evaluation function adapted to the bSSACS-KELM hybrid model is reset, as shown in Eq. (10).


(10) $$\mathrm{fitness}=\alpha \cdot \mathrm{error}+\beta \cdot \frac{\left|R\right|}{\left|D\right|}$$

where α represents a weight that evaluates the significance of classification error rate, error denotes the error rate of the classifier model; β is the number of selected feature, |*R*| denotes the number of attributes in final subset and |*D*| is the dimension of the dataset, i.e., the number of attributes in the entire set. In this work, α = 0.99 and β = 0.01, as set in many previous research.

## 4 Results and discussion

Benchmark function tests are conducted in this part to verify the overall performance of SSACS. Then, to prove the practicability of SSACS on the feature selection problem, experiments based on seven public data sets are conducted in this paper. To better demonstrate the power of the suggested SSACS-KELM model, this paper conducts aided diagnosis experiments on PE data collected from hospitals.

### 4.1 Benchmark functions comparison experiment

To prove the optimization superiority of the SSACS method itself, this subsection uses the classical CEC2014 benchmark test function set for a comprehensive evaluation of the algorithm performance.

#### 4.1.1 Benchmark test experiment setup

First, to make sure the fairness of the comparison experiments of optimization algorithms, following other AI based rules for fair testing (Wu et al., 2018; Yang et al., 2022; Zhang Y. et al., 2022), the initial population number is 30, and the internal parameters of each algorithm are set to default values; in addition, to avoid the contingency of comparison experiments as much as possible, the number of evaluations of algorithms is 30,000, and every approach is repeatedly tested 30 times on this basis. In the analysis of experimental results, the mean value method, Wilcoxon signed-rank test, and Freidman test are employed to ensure the correctness and reliability of the analysis outcomes. Moreover, all experiments are conducted on the same computer, and the software used in the experiments is Matlab2017b, and the core hardware is Intel(R) Xeon(R) CPUE5-2660v3 (2.60 GHz). Eventually, the specific description of the CEC2014 test set is displayed in Table 4.

**TABLE 4:** Description of the 30 benchmark functions.

Class	No.	Functions	${\text{F}}_{i}^{*}={\text{F}}_{i}\,\left({\text{x}}^{*}\right)$
Unimodal functions	1	Rotated high conditioned elliptic function	100
	2	Rotated bent cigar function	200
	3	Rotated discus function	300
Simple multimodal functions	4	Shifted and rotated Rosenbrock’s function	400
	5	Shifted and rotated Ackley’s function	500
	6	Shifted and rotated Weierstrass function	600
	7	Shifted and rotated Griewank’s function	700
	8	Shifted Rastrigin’s function	800
	9	Shifted and rotated Rastrigin’s function	900
	10	Shifted Schwefel’s function	1000
	11	Shifted and rotated Schwefel’s function	1100
	12	Shifted and rotated Katsuura function	1200
	13	Shifted and rotated HappyCat function	1300
	14	Shifted and rotated HGBat function	1400
	15	Shifted and rotated expanded Griewank’s plus Rosenbrock’s function	1500
	16	Shifted and rotated expanded Scaffer’s F6 function	1600
Hybrid functions	17	Hybrid function 1 (*N* = 3)	1700
	18	Hybrid function 2 (*N* = 3)	1800
	19	Hybrid function 3 (*N* = 4)	1900
	20	Hybrid function 4 (*N* = 4)	2000
	21	Hybrid function 5 (*N* = 5)	2100
	22	Hybrid function 6 (*N* = 5)	2200
Composition functions	23	Composition function 1 (*N* = 5)	2300
	24	Composition function 2 (*N* = 3)	2400
	25	Composition function 3 (*N* = 3)	2500
	26	Composition function 4 (*N* = 5)	2600
	27	Composition function 5 (*N* = 5)	2700
	28	Composition function 6 (*N* = 5)	2800
	29	Composition function 7 (*N* = 3)	2900
	30	Composition function 8 (*N* = 3)	3000

#### 4.1.2 Benchmark functions comparison experiment

To validate the superiority performance of SSACS algorithm, SSACS is compared with five original classical algorithms and five improved latest algorithms. Classical algorithms include SSA, CS, SCA, PSO, and MFO. Improved algorithms are made up of CBA, SCADE, SMFO, IGWO, and ACWOA. There are four classes of functions of the test set, including unimodal functions, multimodal functions, hybrid functions, and composition functions. The superiority of SSACS can be demonstrated in all aspects.

Table 5 illustrates the experimental results of benchmark tests, where Avg is the mean and Std denotes the variance of a algorithm after run 30 times, respectively. The best results are bolded in each column. By viewing the mean values from Table 5, it can be seen that SSACS has the smallest mean value for most of benchmark functions, indicating that SSACS achieves relatively higher quality solutions than the others. Variance values obtained by SSACS are smaller than the others, which represents the higher stability of SSACS in optimizing the benchmark functions.

**TABLE 5:** Comparative results of SSACS with peer swarm intelligence algorithms.

	F1		F2		F3	
	Avg	*Std*	Avg	*Std*	Avg	*Std*
SSACS	**1.5305E+05**	**7.6624E+04**	1.0000E+10	**0.0000E+00**	3.0000E+02	4.8692E−07
SSA	1.8596E+06	7.0084E+05	1.0776E+04	1.0603E+04	1.4178E+03	5.9472E+02
CS	1.1978E+06	4.9497E+05	1.0000E+10	0.0000E+00	**3.0000E+02**	**3.3492E**−**09**
SCA	2.3528E+08	6.9501E+07	1.6904E+10	3.0885E+09	3.6036E+04	7.4915E+03
PSO	9.0024E+06	2.7237E+06	1.4685E+08	1.3469E+07	9.3760E+02	1.2161E+02
MFO	1.1091E+08	1.2450E+08	1.1655E+10	8.4305E+09	1.1185E+05	5.2547E+04
CBA	4.5071E+06	1.5824E+06	**9.4105E+03**	7.7486E+03	5.0098E+03	6.5034E+03
SCADE	4.9037E+08	7.1471E+07	3.0125E+10	3.9419E+09	5.6506E+04	6.6783E+03
SMFO	8.1396E+08	2.9141E+08	4.3063E+10	1.1416E+10	7.5643E+04	7.5801E+03
IGWO	1.6657E+07	7.1265E+06	2.1259E+06	9.4622E+05	6.2834E+03	2.3790E+03
ACWOA	1.5760E+08	6.3861E+07	7.1385E+09	3.2777E+09	5.0165E+04	9.4496E+03

	**F4**		**F5**		**F6**	
	**Avg**	***Std***	**Avg**	***Std***	**Avg**	***Std***

SSACS	**4.0454E+02**	**1.2923E+01**	**5.2004E+02**	5.7788E−02	**6.1527E+02**	4.2093E+00
SSA	4.8757E+02	3.6108E+01	5.2006E+02	5.8801E−02	6.1845E+02	3.8175E+00
CS	4.2726E+02	3.2623E+01	5.2083E+02	5.7655E−02	6.2483E+02	**1.6563E+00**
SCA	1.4322E+03	2.5735E+02	5.2094E+02	**4.2208E**−**02**	6.3325E+02	2.3141E+00
PSO	4.7275E+02	4.3189E+01	5.2094E+02	4.2282E−02	6.2221E+02	2.9204E+00
MFO	1.3930E+03	8.5099E+02	5.2024E+02	1.9434E−01	6.2467E+02	3.3657E+00
CBA	4.9837E+02	3.3237E+01	5.2014E+02	1.9687E−01	6.3941E+02	3.0695E+00
SCADE	2.2667E+03	4.8950E+02	5.2096E+02	4.3342E−02	6.3432E+02	2.4224E+00
SMFO	7.0204E+03	2.6299E+03	5.2090E+02	8.6576E−02	6.3836E+02	2.1000E+00
IGWO	5.2014E+02	3.1220E+01	5.2053E+02	1.5103E−01	6.1946E+02	2.8638E+00
ACWOA	1.2191E+03	4.6089E+02	5.2080E+02	1.6444E−01	6.3324E+02	2.2305E+00

	**F7**		**F8**		**F9**	
	**Avg**	***Std***	**Avg**	***Std***	**Avg**	***Std***

SSACS	**7.0000E+02**	**9.0641E**−**09**	**8.2168E+02**	7.1351E+00	**1.0045E+03**	1.8697E+01
SSA	7.0001E+02	1.0224E−02	9.0744E+02	2.7149E+01	1.0075E+03	2.5665E+01
CS	7.0000E+02	1.8016E−03	8.3010E+02	**5.4559E+00**	1.0409E+03	2.2051E+01
SCA	8.3328E+02	3.7302E+01	1.0416E+03	2.8526E+01	1.1708E+03	1.9179E+01
PSO	7.0232E+02	1.6743E−01	9.7051E+02	2.3160E+01	1.1110E+03	2.6577E+01
MFO	7.9998E+02	6.7237E+01	9.3824E+02	4.0258E+01	1.1056E+03	5.1840E+01
CBA	7.0001E+02	1.3192E−02	1.0112E+03	4.9803E+01	1.1633E+03	6.2608E+01
SCADE	8.8714E+02	3.6260E+01	1.0699E+03	1.9488E+01	1.2067E+03	**1.7405E+01**
SMFO	1.0074E+03	8.7666E+01	1.0779E+03	2.8079E+01	1.2171E+03	2.0380E+01
IGWO	7.0096E+02	9.0343E−02	8.8963E+02	1.9110E+01	1.0153E+03	2.4640E+01
ACWOA	7.3772E+02	2.3945E+01	9.9219E+02	2.2500E+01	1.1347E+03	2.4153E+01

	**F10**		**F11**		**F12**	
	**Avg**	***Std***	**Avg**	***Std***	**Avg**	***Std***

SSACS	**1.7818E+03**	2.7743E+02	**4.1829E+03**	2.9480E+02	**1.2001E+03**	**4.1058E**−**02**
SSA	4.3706E+03	6.6190E+02	4.8537E+03	7.3362E+02	1.2004E+03	2.0277E−01
CS	2.0560E+03	**2.4299E+02**	4.6119E+03	3.0117E+02	1.2008E+03	1.0627E−01
SCA	6.9709E+03	4.7807E+02	8.0593E+03	3.4507E+02	1.2024E+03	2.8540E−01
PSO	5.1984E+03	5.8340E+02	5.8494E+03	6.1109E+02	1.2025E+03	2.1388E−01
MFO	4.4727E+03	8.6847E+02	5.2184E+03	7.1480E+02	1.2005E+03	2.2144E−01
CBA	5.4694E+03	7.2200E+02	5.8944E+03	6.1739E+02	1.2011E+03	4.2208E−01
SCADE	7.2395E+03	3.9331E+02	8.1839E+03	**2.5706E+02**	1.2026E+03	3.0303E−01
SMFO	7.4697E+03	4.6484E+02	8.2682E+03	4.9074E+02	1.2023E+03	3.9524E−01
IGWO	3.5766E+03	6.0607E+02	4.6287E+03	6.4620E+02	1.2007E+03	3.0636E−01
ACWOA	4.8013E+03	7.8277E+02	6.3415E+03	7.2310E+02	1.2018E+03	5.4145E−01

	**F13**		**F14**		**F15**	
	**Avg**	***Std***	**Avg**	***Std***	**Avg**	***Std***

SSACS	**1.3002E+03**	**5.0972E**−**02**	**1.4002E+03**	3.2542E−02	**1.5064E+03**	1.8784E+00
SSA	1.3005E+03	1.1367E−01	1.4004E+03	2.4055E−01	1.5093E+03	3.1472E+00
CS	1.3003E+03	5.5667E−02	1.4002E+03	**2.9906E**−**02**	1.5111E+03	2.3256E+00
SCA	1.3029E+03	3.4656E−01	1.4442E+03	7.2420E+00	4.5504E+03	2.1786E+03
PSO	1.3004E+03	8.0161E−02	1.4003E+03	1.2221E−01	1.5163E+03	**1.4908E+00**
MFO	1.3021E+03	1.3630E+00	1.4323E+03	2.3698E+01	1.0120E+05	1.5193E+05
CBA	1.3005E+03	1.3003E−01	1.4003E+03	1.3822E−01	1.5673E+03	1.7886E+01
SCADE	1.3039E+03	1.9797E−01	1.4861E+03	1.3544E+01	1.7835E+04	7.6600E+03
SMFO	1.3054E+03	1.0109E+00	1.5372E+03	4.1593E+01	5.1691E+04	4.5347E+04
IGWO	1.3006E+03	1.0192E−01	1.4003E+03	2.0992E−01	1.5159E+03	4.7386E+00
ACWOA	1.3017E+03	1.0026E+00	1.4166E+03	1.1640E+01	1.9832E+03	5.8631E+02

	**F16**		**F17**		**F18**	
	**Avg**	***Std***	**Avg**	***Std***	**Avg**	***Std***

SSACS	**1.6115E+03**	6.1321E−01	**3.3120E+03**	4.9144E+02	**1.8710E+03**	2.6342E+01
SSA	1.6117E+03	7.6459E−01	1.3223E+05	9.4160E+04	5.4251E+03	2.8516E+03
CS	1.6124E+03	1.9734E−01	3.8911E+03	**2.4956E+02**	1.8732E+03	**1.7226E+01**
SCA	1.6127E+03	2.7115E−01	6.2377E+06	3.1462E+06	1.7361E+08	8.4804E+07
PSO	1.6120E+03	5.3353E−01	2.5217E+05	9.7430E+04	2.0452E+06	5.0246E+05
MFO	1.6129E+03	5.7196E−01	2.9230E+06	3.6385E+06	2.5133E+07	1.3716E+08
CBA	1.6133E+03	3.3415E−01	2.3316E+05	1.7157E+05	6.6000E+03	4.6311E+03
SCADE	1.6128E+03	**1.5927E**−**01**	1.4708E+07	7.0964E+06	1.8190E+08	1.1361E+08
SMFO	1.6126E+03	2.6648E−01	4.3572E+07	4.3075E+07	7.2172E+08	6.4484E+08
IGWO	1.6116E+03	6.2946E−01	9.6883E+05	6.8163E+05	2.1875E+04	3.2060E+04
ACWOA	1.6122E+03	5.2104E−01	2.1762E+07	1.3663E+07	5.2842E+07	4.8926E+07

	**F19**		**F20**		**F21**	
	**Avg**	***Std***	**Avg**	***Std***	**Avg**	***Std***

SSACS	**1.9064E+03**	**7.4411E**−**01**	**2.0500E+03**	1.7401E+01	**2.9571E+03**	**1.9740E+02**
SSA	1.9141E+03	2.1321E+00	2.3600E+03	8.7168E+01	5.7097E+04	4.8479E+04
CS	1.9079E+03	9.6224E−01	2.0558E+03	**1.3542E+01**	3.0697E+03	2.1758E+02
SCA	1.9864E+03	2.0455E+01	1.6212E+04	5.9168E+03	1.3332E+06	6.0373E+05
PSO	1.9178E+03	2.7564E+00	2.3379E+03	6.6419E+01	1.1894E+05	6.5836E+04
MFO	1.9633E+03	5.2391E+01	1.0044E+05	1.2069E+05	2.1203E+06	7.4718E+06
CBA	1.9321E+03	3.2419E+01	3.1634E+03	1.2995E+03	1.1892E+05	7.5593E+04
SCADE	2.0132E+03	1.3645E+01	2.4525E+04	6.9326E+03	2.4411E+06	1.5081E+06
SMFO	2.1704E+03	8.5390E+01	9.7620E+04	1.2569E+05	2.3357E+07	1.9225E+07
IGWO	1.9163E+03	2.8444E+00	3.9006E+03	1.9533E+03	3.5076E+05	2.6508E+05
ACWOA	2.0129E+03	4.2759E+01	3.1689E+04	1.3314E+04	4.4990E+06	3.4834E+06

	**F22**		**F23**		**F24**	
	**Avg**	***Std***	**Avg**	***Std***	**Avg**	***Std***

SSACS	**2.3956E+03**	**9.4518E+01**	2.6152E+03	9.2504E−13	2.6249E+03	1.2091E+00
SSA	2.5956E+03	1.6596E+02	2.6153E+03	1.5908E−02	2.6409E+03	7.7351E+00
CS	2.4520E+03	9.7415E+01	2.6152E+03	1.3747E−12	2.6256E+03	1.1883E+00
SCA	2.9426E+03	1.2026E+02	2.6689E+03	1.1485E+01	2.6001E+03	8.2194E−02
PSO	2.8574E+03	2.3362E+02	2.6161E+03	5.7559E−01	2.6269E+03	6.7535E+00
MFO	3.0401E+03	3.4313E+02	2.6878E+03	5.6193E+01	2.6760E+03	2.9128E+01
CBA	3.3602E+03	3.1116E+02	2.6158E+03	2.7425E−01	2.6846E+03	3.4466E+01
SCADE	3.0688E+03	1.6394E+02	**2.5000E+03**	**0.0000E+00**	**2.6000E+03**	**2.3118E**−**06**
SMFO	3.4814E+03	6.4031E+02	2.5000E+03	0.0000E+00	2.6000E+03	4.8541E−06
IGWO	2.5964E+03	1.7419E+02	2.6224E+03	3.2919E+00	2.6000E+03	5.7849E−03
ACWOA	3.1264E+03	2.3408E+02	2.5325E+03	7.4023E+01	2.6000E+03	1.3236E−05

	**F25**		**F26**		**F27**	
	**Avg**	***Std***	**Avg**	***Std***	**Avg**	***Std***

SSACS	2.7044E+03	1.0596E+00	**2.7002E+03**	6.4524E−02	3.1019E+03	7.5526E−01
SSA	2.7116E+03	4.1282E+00	2.7006E+03	1.2918E−01	3.4663E+03	1.6607E+02
CS	2.7057E+03	1.3162E+00	2.7003E+03	**6.1900E**−**02**	3.1087E+03	6.3116E+00
SCA	2.7251E+03	8.2339E+00	2.7025E+03	6.0714E−01	3.4946E+03	3.2336E+02
PSO	2.7126E+03	5.5087E+00	2.7872E+03	3.4612E+01	3.4272E+03	3.0759E+02
MFO	2.7164E+03	1.1278E+01	2.7023E+03	1.4525E+00	3.6674E+03	1.8792E+02
CBA	2.7365E+03	1.6057E+01	2.7106E+03	5.5105E+01	4.0093E+03	4.1839E+02
SCADE	**2.7000E+03**	**0.0000E+00**	2.7037E+03	4.9502E−01	3.1970E+03	2.3009E+02
SMFO	2.7000E+03	0.0000E+00	2.7491E+03	4.5363E+01	**2.9000E+03**	**0.0000E+00**
IGWO	2.7097E+03	2.3386E+00	2.7007E+03	1.6078E−01	3.1107E+03	5.1704E+00
ACWOA	2.7000E+03	0.0000E+00	2.7438E+03	4.9984E+01	3.6797E+03	3.7660E+02

	**F28**		**F29**		**F30**	
	**Avg**	***Std***	**Avg**	***Std***	**Avg**	***Std***

SSACS	3.7288E+03	7.0830E+01	3.9240E+03	1.2958E+02	**4.7588E+03**	**3.6568E+02**
SSA	3.8593E+03	2.8295E+02	3.0717E+06	5.2359E+06	1.1678E+04	3.3452E+03
CS	3.7717E+03	5.1184E+01	**3.9001E+03**	**9.4392E+01**	4.7947E+03	3.6983E+02
SCA	4.8548E+03	3.6287E+02	9.9182E+06	5.0267E+06	2.6991E+05	9.9071E+04
PSO	6.9150E+03	7.5970E+02	2.6824E+04	5.2180E+04	1.3278E+04	5.5336E+03
MFO	3.9130E+03	1.5820E+02	2.4916E+06	3.2052E+06	5.6241E+04	5.2002E+04
CBA	5.4492E+03	8.2514E+02	3.5947E+07	4.3112E+07	2.9730E+04	5.4889E+04
SCADE	5.3278E+03	4.7543E+02	1.7381E+07	1.0264E+07	4.3698E+05	1.5944E+05
SMFO	3.0000E+03	**0.0000E+00**	7.4962E+05	4.0889E+06	7.5821E+05	7.1805E+05
IGWO	3.8590E+03	1.5799E+02	1.7487E+06	4.4972E+06	2.8804E+04	1.2917E+04
ACWOA	4.1662E+03	1.2071E+03	2.0752E+07	1.7503E+07	3.9836E+05	3.3714E+05

**FIGURE 3:**
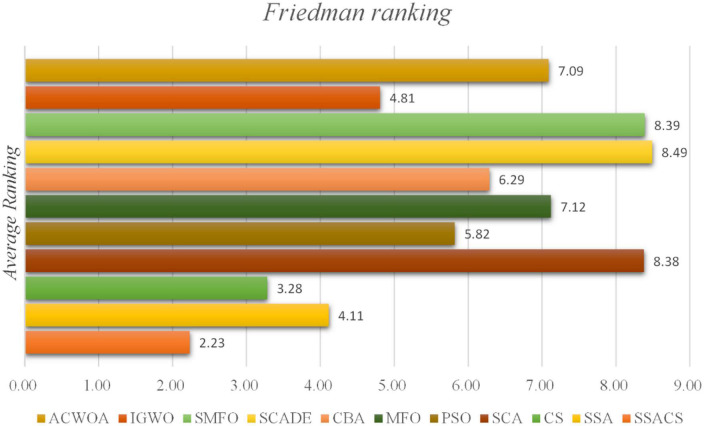
Friedman test results.

In order to more accurately analyze the experimental outcomes of the benchmark function of SSACS, Table 6 shows the test results of the Wilcoxon signed-rank test, where ‘+/−/=’ represents the performance “better than other algorithms/worse than other algorithms/equal to other algorithms,” ‘Mean-level’ denotes the average ranking of 30 replicate experiments, and ‘rank’ denotes the final overall ranking. It can be seen that SSACS outperforms at least 21 benchmark functions compared with all other 8 algorithms, and the overall ranking of SSACS is in first place, which verifies that the above experimental results are correct. In addition, the average ranking of SSACS is also far ahead compared to the second place, which also verifies the high stability of the algorithm. To further prove the correctness and credibility of the test outcomes, the benchmark test outcomes of SSACS are also analyzed using the Freidman test method. It can be seen that in Figure 3 Freidman test, SSACS also ranks first, far ahead of other comparison algorithms. Therefore, these two tests can prove that the benchmark function experimental results of SSACS at this time are credible and accurate, and the performance of SSACS is also very strong.

**TABLE 6:** Wilcoxon signed-rank results.

Algorithm	+/−/=	Mean-level	Rank
**SSACS**	**∼**	**1.67**	**1**
SSA	26/1/3	3.97	3
CS	21/1/8	3.10	2
SCA	29/1/0	8.23	9
PSO	27/1/2	5.77	5
MFO	29/0/1	7.37	8
CBA	28/1/1	6.77	6
SCADE	26/3/1	8.50	10
SMFO	25/5/0	8.63	11
IGWO	26/2/2	4.60	4
ACWOA	26/4/0	7.20	7

**FIGURE 4:**
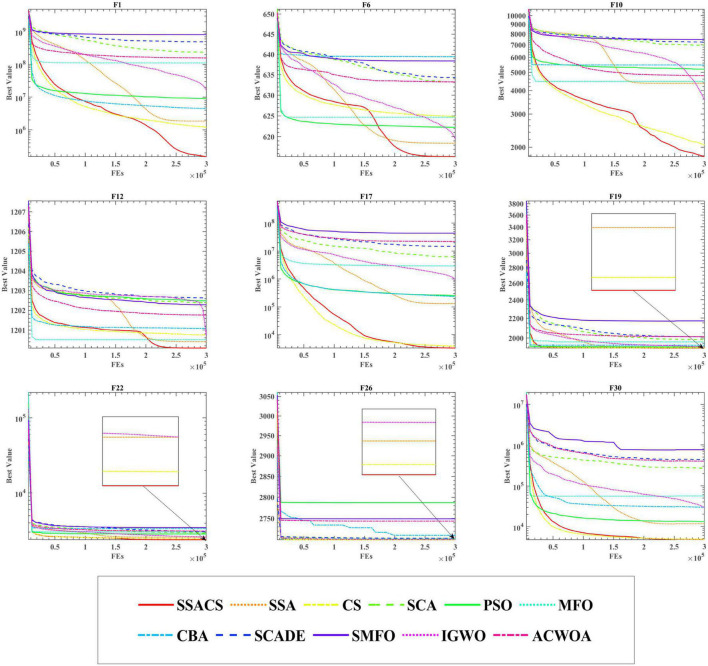
Convergence curves of SSACS and competitors.

To show the advantages of SSACS over other peer algorithms in more detail, this paper also records the change process of the values of the optimal solutions generated by SSACS iterations, as shown in Figure 4. In F1 for Unimodal Functions, SSACS converges with higher accuracy in the middle and late phases of the iteration, which indicates that it has better optimization performance for simple optimization problems. In F6, F10, and F12 of Simple Multimodal Functions, SSACS can successfully get rid of LO during the iteration, which indicates that it has a stronger global optimization ability on multi-peaked problems. In F19, F22, F26, and F30 of Hybrid Functions and Composition Functions, SSACS has stronger exploitation ability and convergence speed, which represents that it has higher optimality finding efficiency when dealing with complex problems. In summary, SSACS is a very excellent group intelligence optimization algorithm.

### 4.2 Feature selection experiments

In this part, first, the evaluation criteria for the feature selection experiments are described. Immediately after, SSACS is used in feature selection experiments on public datasets to make clear the efficiency and generalization of SSACS to handle feature selection problems. Finally, SSACS is used in a real PE problem to select the five key-features.

#### 4.2.1 Feature selection experimental setup

In the feature selection experiment, the same experimental environment of the benchmark function is followed, and in particular, five different feature selection evaluation metrics are added. The details are described below.

The confusion matrix is a schematic diagram for recording classification prediction results in the field of pattern recognition, which describes the relationship between the true category attributes of sample data and the predicted category and is a significant indicator for assessing the performance of classification models. Evaluation metrics such as accuracy, specificity, and sensitivity can be calculated using the confusion matrix. The binary classification problem divides the samples into positive and negative samples. The confusion matrix consists of True Positives (TP), True Negatives (TN), False Positives (FP), and False Negatives (FP).

Accuracy (ACC) is the most commonly used classification evaluation metric, which measures the classifier’s ability to recognize the correct samples. The accuracy range is located in [0, 1], and the closer the accuracy is to 1, the better the classification performance of the classifier is. The accuracy rate is calculated as follows.


(11) $$A\,C\,C=\frac{T\,P+T\,N}{T\,P+T\,N+F\,P+F\,N}$$

Precision (PRE) dictates the proportion of the examples classified as positive cases that are actually positive cases. Similarly, the closer the precision is to 1 means the better the classifier is, and the precision is calculated as follows.


(12) $$P\,r\,e\,c\,i\,s\,i\,o\,n=\frac{T\,P}{T\,P+F\,P}$$

Sensitivity (SEN) is the probability that a patient is actually ill and is diagnosed as such, and it measures the classifier’s ability to identify positive cases. The closer the sensitivity is to 1, the better the ability to test patients. The sensitivity is calculated as follows.


(13) $$S\,e\,n\,s\,i\,t\,i\,v\,i\,t\,y=\frac{T\,P}{T\,P+F\,N}$$

Specificity (SPE) is the probability that an actual disease-free individual is diagnosed as disease-free, and it measures the ability of the classifier to recognize negative cases, reflecting the ability of the classifier to identify disease-free individuals. Specificity is calculated as follows.


(14) $$S\,p\,e\,c\,i\,f\,i\,c\,i\,t\,y=\frac{T\,N}{T\,N+F\,N}$$

MCC reflects the correlation between diagnostic results and actual outcomes. For balanced data, higher ACC and MCC values both point to higher quality predictions, while for unbalanced data, MCC is a more accurate reflection of the predictor’s predictive quality than ACC. MCC is calculated as follows.


(15) $$M\,C\,C=\frac{T\,P\times T\,N-F\,P\times F\,N}{\sqrt{\begin{array}{c} \left(T\,P+F\,P\right)\times \left(T\,P+F\,N\right)\\ \times \left(T\,N+F\,P\right)\times \left(T\,N+F\,N\right) \end{array}}}$$

In addition to the classification evaluation method, the discrete algorithms re-used in this experiment include bSSACS, bMFO, bGWO, BGSA, BPSO, bALO, BBA, BSSA, bWOA, and bCS. Table 7 displays the values of the key parameters set for each algorithm. In addition, the number of populations for the feature selection experiments is set to 20, and the experimental results are validated using the classical ten-fold cross-validation method.

**TABLE 7:** Parameter setting of the optimization algorithms.

Algorithms	bSSACS	bMFO	bGWO	BGSA	BPSO
Values	*pa = 0.25*	*a = 2, b = 1*	*a* = [0,2]	*wMax = 20; wmin = 1e-10*	*Max = 0.9, min = 0.4*

**Algorithms**	**bALO**	**BBA**	**BSSA**	**bWOA**	**bCS**

Values	∼	*a = 0.5; r = 0.5*	∼	*a* = [0,2]	*pa = 0.25*

#### 4.2.2 Public dataset experiments

We must evaluate algorithmic features in computer science to assess the influence of computational pieces since diverse strategies depend on different traits and trends (Cao et al., 2022; Liu Y. et al., 2022; Zhang Z. et al., 2022). In the part, to prove the feature selection practicality of bSSACS-KELM against different datasets, 8 public datasets from the well-known machine learning database UCI Machine Learning Repository are used in this paper. Table 8 shows the key parameters of these datasets. In order to distinguish these feature datasets as much as possible to validate the performance of bSSACS-KELM accurately, the datasets used in this paper have a relatively large difference in the number of samples and features. The largest number of samples in the dataset is 569 in BreastEW, and the smallest is 152 in JPNdata; the largest number of features is 30, and the smallest is 10. Finally, the public dataset experiments also use the mean and variance to represent the experimental results accurately. In addition, mean and total ranking is used in this paper to show the superiority of bSSACS-KELM in different datasets visually.

**TABLE 8:** The 8 public-datasets.

Datasets	Samples	Features	Classes
Hepatitis	155	19	2
BreastEW	569	30	2
JPNdata	152	10	2
Heart	270	14	2
IonosphereEW	351	34	2
CongressEW	435	16	2
HeartEW	270	27	2
wdbc	560	30	2

Table 9 shows detailed data on the Accuracy of bSSACS-KELM compared with other famous methods. It can be observed that bSSACS-KELM has the highest accuracy in most of the datasets, with the highest being 98.8% for wdbc. In addition, the lowest accuracy of bSSACS-KELM is 93%. The combination of the highest and lowest values means that the classification performance of bSSACS-KELM is better in terms of accuracy and stability, regardless of the dataset. In contrast, other similar methods, especially the unimproved CS-KELM and SSA-KELM, are far inferior to bSSACS-KELM. Table 10 displays the average and overall ranking of bSSACS with other approaches on different datasets, and the results show that in agreement with the experimental analysis, bSSACS ranks first.

**TABLE 9:** Accuracy results of experiments on public datasets.

	Hepatitis		BreastEW		wdbc		JPNdata	
	AVE	*STD*	AVE	*STD*	AVE	*STD*	AVE	*STD*
bMFO	0.948	0.051	0.986	0.023	0.982	0.022	0.902	0.084
bGWO	0.961	0.045	0.986	0.014	0.982	0.014	0.889	**0.054**
BGSA	0.948	0.051	**0.988**	0.017	0.984	0.013	0.895	0.063
bALO	0.864	0.072	0.954	0.028	0.958	0.021	0.703	0.138
BBA	0.852	0.060	0.960	0.025	0.954	0.025	0.700	0.149
BSSA	0.948	0.040	0.984	0.022	0.981	**0.010**	0.920	0.077
bWOA	**0.968**	**0.034**	0.988	0.012	0.979	0.016	0.894	0.086
bCS	0.911	0.041	0.974	0.022	0.972	0.015	0.882	0.059
bSSACS	**0.968**	**0.034**	**0.988**	**0.008**	**0.986**	0.011	**0.933**	0.056

	**Heart**		**IonosphereEW**		**CongressEW**		**HeartEW**	
	**AVE**	**STD**	**AVE**	**STD**	**AVE**	**STD**	**AVE**	**STD**

bMFO	0.933	0.049	0.963	0.019	**0.975**	0.025	0.933	0.052
bGWO	0.922	**0.032**	0.963	0.030	0.972	0.026	0.941	0.040
BGSA	0.930	0.056	**0.966**	**0.019**	0.968	0.029	**0.944**	**0.020**
bALO	0.822	0.072	0.855	0.039	0.939	0.049	0.841	0.058
BBA	0.815	0.105	0.900	0.050	0.926	0.050	0.826	0.078
BSSA	**0.937**	0.043	0.957	0.028	0.968	0.031	0.937	0.053
bWOA	0.922	0.037	0.963	0.038	0.972	**0.021**	0.930	0.044
bCS	0.881	0.055	0.937	0.044	0.959	0.034	0.896	0.049
bSSACS	0.930	0.044	0.958	0.033	**0.975**	0.025	0.937	0.043

**TABLE 10:** The ranking of bSSACS-KELM and its peer methods.

Methods	bMFO	bGWO	BGSA	bALO	BBA	BSSA	bWOA	bCS	bSSACS
Average-ranking	3.375	3.75	2.625	8.25	8.75	4.125	4	7	**2.125**
Overall-ranking	3	4	2	8	9	6	5	7	**1**

To further demonstrate the superiority of bSSACS-KELM in feature selection, the classification results were carefully evaluated using SEN. Table 11 shows the outcomes of this Sensitivity evaluation, and Table 12, indicates the Sensitivity ranking results. It can be seen that bSSACS performs equally well in this data, with bSSACS ranking first and bALO and BBA ranking last. Finally, after different datasets and different evaluation methods, experiments prove that bSSACS-KELM has good feature classification performance.

**TABLE 11:** Sensitivity results of experiments on public datasets.

	Hepatitis		BreastEW		wdbc		JPNdata	
	AVE	*STD*	AVE	*STD*	AVE	*STD*	AVE	*STD*
bMFO	0.758	0.237	**1.000**	**0.000**	0.952	0.059	**0.961**	0.087
bGWO	0.817	0.200	**1.000**	**0.000**	0.953	0.038	0.930	0.138
BGSA	0.758	0.237	**1.000**	**0.000**	0.958	0.034	0.946	**0.069**
bALO	0.475	0.360	0.978	0.037	0.886	0.056	0.621	0.370
BBA	0.417	0.236	0.997	0.009	0.882	0.060	0.786	0.160
BSSA	0.775	0.222	**1.000**	**0.000**	0.948	**0.026**	0.877	0.105
bWOA	**0.842**	**0.169**	**1.000**	**0.000**	0.943	0.044	0.934	0.070
bCS	0.642	0.242	0.997	0.009	0.924	0.040	0.948	0.089
bSSACS	**0.842**	**0.169**	**1.000**	**0.000**	**0.962**	0.030	0.934	0.070

	**Heart**		**IonosphereEW**		**CongressEW**		**HeartEW**	
	**AVE**	***STD***	**AVE**	***STD***	**AVE**	***STD***	**AVE**	***STD***

bMFO	0.960	0.047	**1.000**	**0.000**	**0.970**	0.039	0.960	0.056
bGWO	0.947	0.042	**1.000**	**0.000**	0.962	0.041	0.927	0.049
BGSA	0.960	0.056	**1.000**	**0.000**	0.959	0.051	**0.967**	0.047
bALO	0.867	0.054	0.951	0.015	0.924	0.063	0.860	0.066
BBA	0.860	0.086	0.982	0.031	0.910	0.052	0.893	0.095
BSSA	0.960	0.034	0.991	0.027	0.963	0.035	0.960	**0.034**
bWOA	0.953	0.063	**1.000**	**0.000**	0.963	**0.030**	0.960	0.047
bCS	0.927	0.049	0.991	0.019	0.944	0.047	0.927	0.058
bSSACS	**0.987**	**0.028**	**1.000**	**0.000**	0.963	0.039	**0.967**	0.035

**TABLE 12:** The ranking of bSSACS-KELM and its peer methods.

Methods	bMFO	bGWO	BGSA	bALO	BBA	BSSA	bWOA	bCS	bSSACS
Average-ranking	2.25	3.875	2.625	8.5	8.5	3.75	2.875	6.25	**1.5**
Overall-ranking	2	6	3	8	8	5	4	7	**1**

### 4.3 Feature selection experiment in PE dataset

In this section, to prove the practicality of the bSSACS-KELM model to process real data from hospitals and to assist realistic doctors in diagnosing diseases, this paper uses PE datasets collected from hospitals for classification prediction. First, to further demonstrate that the combination of bSSACS and KELM is excellent, this experiment combines bSSACS with four different classifiers, including FKNN, KNN, MLP, and SVM, with the help of the PE dataset for comparison experiments. Then, to show the performance difference between bSSACS-KELM and classical methods, this experiment is designed to compare bSSACS with five classical methods, including BP, CART, RandomF, and so on, where the algorithm settings and codes are from MATLAB default. Furthermore, to illustrate the advantages of bSSACS over other algorithms of the same type, this experiment is designed to compare bSSACS with nine other swarm intelligence algorithms, including bSSA, bALO, and bCS, etc. For the purpose of more accurately assessing the processing capability of bSSACS-KELM model on real data, four evaluation methods, including Accuracy, Precision, Specificity, and MCC, will be used in this section of the experiment to illustrate the reliability of the classification results fully. Finally, the results of the experiments, after 10-fold cross-validation, yield the five most critical features of the PE dataset.

**FIGURE 5:**
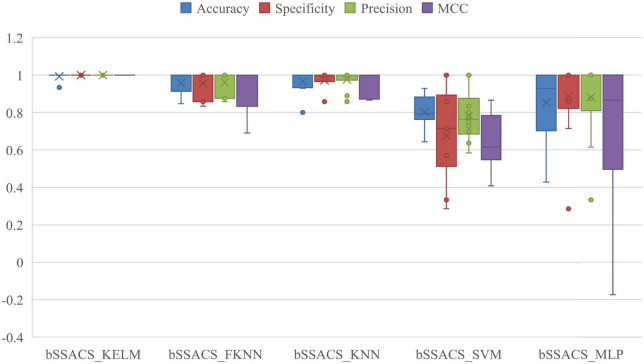
Results of five different classifiers based on SSACS.

The combination of different classifiers of bSSACS will have different classification effects. To demonstrate that the combination of bSSACS and KELM is excellent, bSSACS was combined with four other classifiers, and a comparison experiment was conducted, and the outcomes are displayed in Figure 5. It can be viewed that the results of bSSACS-KELM are far ahead of the other classifiers in the four aspects of Accuracy, Precision, Specificity, and the box plot can also know MCC, and the stability of the KELM model. In contrast, the combination of bSSACS with SVM and MLP is not accurate enough, and the stability is very poor. Therefore, it can be concluded that the combination of bSSACS and KELM is very suitable.

**FIGURE 6:**
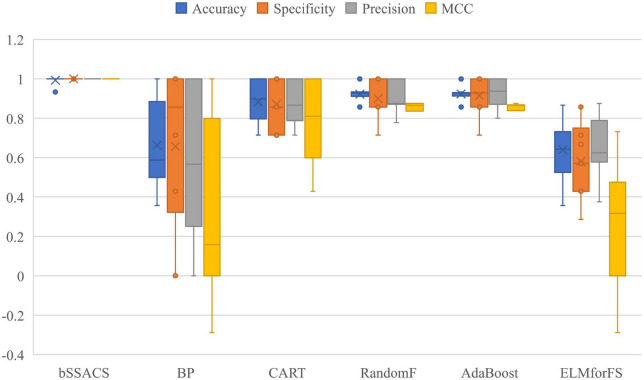
Comparison results of SSACS-KELM with other classical methods.

To further illustrate the benefits of bSSACS-KELM over the performance of classical classification methods, a comparison experiment between bSSACS and classical methods was conducted in this paper. The box plot results of the experiments are shown in Figure 6. It can be seen that bSSACS-KELM has a significant advantage over other classification methods. CART occasionally works well but is much less stable than bSSACS-KELM; the two methods, BP and ELMforFS, have inferior overall performance. The complete test outcomes indicate that bSSACS-KELM has a considerable advantage over the classical classification methods.

**FIGURE 7:**
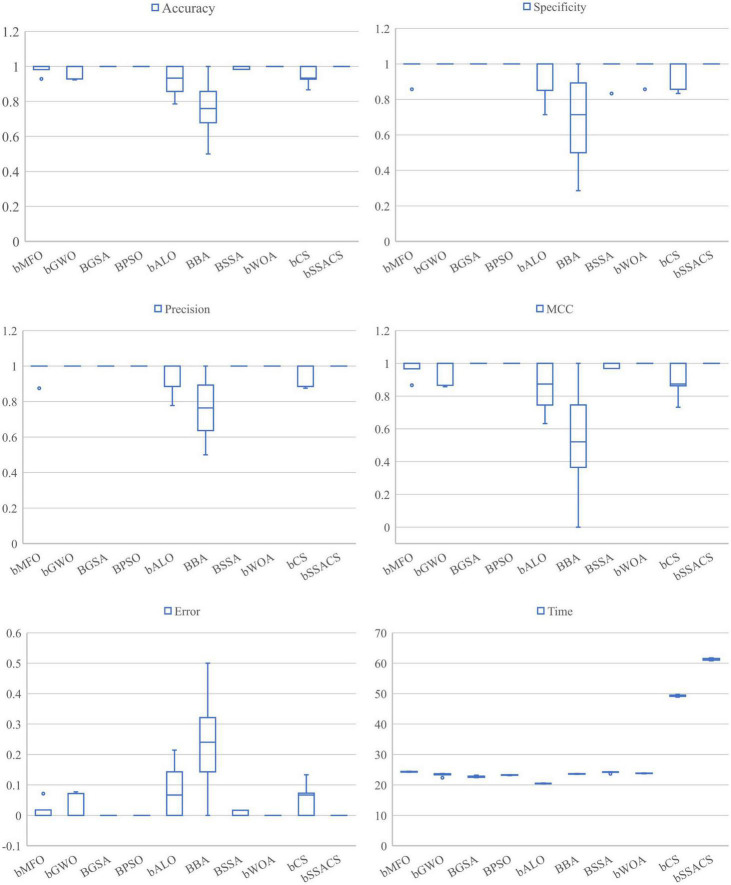
Comparison of SSACS and eight algorithms on six evaluation criteria.

The above experiments can prove that the combination of KELM with the swarm intelligence algorithm will have a stronger performance. To prove the superiority of bSSACS combined with KELM over other competitors, this paper compares the classification models of bSSACS-KELM with other famous SIAs, including BMFO, BGWO, BGSA, BPSO, BALO, BBA, BSSA, and BWOA. Figure 7 box plots represent the outcomes of this comparison experiment. It can be observed that the bSSACS data are close to 1 for the first five classification evaluation metrics, which indicates that the bSSACS-KELM model also has very strong prediction performance among similar algorithms and is well suited for predicting PE problems. The SSACS-KELM has higher accuracy and excellent stability than the original SSA and CS algorithms. Among Accuracy and Error, the integrated classification based on the bSSACS model is better and has the highest accuracy and stability. In Specificity and Precision, the bSSACS model based on the bSSACS model has the highest accuracy for selecting both negative and as positive cases. The correlation between the true and predicted values based on the bSSACS model is the highest in MCC. In Time, it can be seen that bSSACS has an acceptable level of time complexity while improving performance.

To further enhance the credibility of this comparison experiment, this paper uses the Friedman test method to verify and rank the test outcomes, as shown in Table 13. The analysis of the test method shows that bSSACS-KELM is indeed stable in the first place. Therefore, after the above experiments, it can be shown that bSSACS-KELM is a very suitable model for PE-assisted prediction and can effectively classify the PE dataset.

**TABLE 13:** Friedman test results.

Method		bMFO	bGWO	BGSA	BPSO	bALO	BBA	BSSA	bWOA	bCS	bSSACS
Accuracy	Avg	4.85	5.4	4.35	4.45	7.05	9.5	4.55	4.3	6.35	4.2
	Rank	6	7	3	4	9	10	5	2	8	1
Error	Avg	4.85	5.4	4.35	4.45	7.05	9.5	4.55	4.3	6.35	4.2
	Rank	6	7	3	4	9	10	5	2	8	1
Specificity	Avg	4.95	4.95	4.95	4.95	6.15	8.8	4.95	4.95	5.85	4.5
	Rank	2	2	2	2	9	10	2	2	8	1
Precision	Avg	5	5	4.95	5	5.9	9.05	4.95	4.9	5.75	4.5
	Rank	5	5	3	5	9	10	3	2	8	1
MCC	Avg	4.85	5.4	4.35	4.45	6.95	9.5	4.55	4.35	6.45	4.15
	Rank	6	7	2	4	9	10	5	2	8	1
Timecost	Avg	7.6	4.3	2.2	3	1	4.8	7.3	5.8	9	10
	Rank	8	4	2	3	1	5	7	6	9	10

Table 14 shows the detailed data of the experiments on the PE dataset based on the bSSACS-KELM model. The first column is the label of the ten-fold cross-validation, the second one is the number of features selected for feature selection, and the rest are Accuracy, Specificity, Precision, and MCC used above. The value of Accuracy under ten-fold cross-validation is 99.33%, the value of MCC is 0.9875, and the values of Specificity and Precision is 1. Again, this shows that combining bSSACS with KELM turns out to be a very good PE classifier model.

**TABLE 14:** The detailed results acquired by SSACS.

Fold	Selected feature subset size	Accuracy	Specificity	Precision	MCC
#1	13	1.0000	1.0000	1.0000	1.0000
#2	11	1.0000	1.0000	1.0000	1.0000
#3	12	0.9333	1.0000	1.0000	0.8750
#4	10	1.0000	1.0000	1.0000	1.0000
#5	12	1.0000	1.0000	1.0000	1.0000
#6	10	1.0000	1.0000	1.0000	1.0000
#7	8	1.0000	1.0000	1.0000	1.0000
#8	13	1.0000	1.0000	1.0000	1.0000
#9	7	1.0000	1.0000	1.0000	1.0000
#10	11	1.0000	1.0000	1.0000	1.0000
Avg.	∼	**0.9933**	**1.0000**	**1.0000**	**0.9875**
*Std*.	∼	0.0200	0.0000	0.0000	0.0375

**FIGURE 8:**
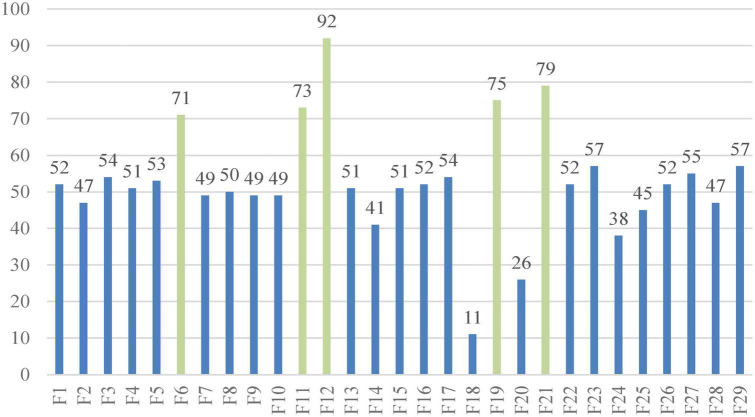
Selected features of the SSACS method.

Figure 8 shows the number of times every indicator was chosen by the bSSACS-KELM model under 10 times 10-fold cross-validation, with the horizontal axis representing the 29 features of the PE dataset and the vertical axis representing the number of times they were selected. The top 5 features are F6, F11, F12, F19, and F21, which represent Syncope, HOT, SBP, D-D, and ALB, respectively, and these five features were selected more often than the other features, which is also consistent with the actual medical statistics.

## 5 Discussion

This study uses the bSSACS-KELM model for feature selection and classification prediction on real PE datasets. First, experiments demonstrate that bSSACS-KELM has higher accuracy and stability than bSSACS-FKNN, bSSACS-KNNN, and bSSACS-MLP models, so that bSSACS-KELM is more suitable and satisfied than the other methods for feature selection of PE datasets. Subsequently, the bSSACS-KELM model is compared to BP and CART. Therefore, bSSACS-KELM has obvious advantages to settle the classification problems. Besides, we experimentally compared bSSACS-KELM with its counterparts BMFO-KELM and bALO-KELM. The test outcomes indicate that bSSACS-KELM has a greater advantage in the same kind of SIAs. In a word, the features chosen by bSSACS-KELM from the PE dataset are statistically summarized, and Syncope, HOT, SBP, D-D, and ALB are identified as the crucial features in the dataset. Previous related work is referred to and analyzed to confirm that the chosen features are consistent with medical statistics.

Early diagnosis plays a critical role in the treatment and prognosis of patients due to the high morbidity and mortality of PE. One or more risk factors of PE, which are non-specific in clinical diagnosis, hinder the progress of diagnosis and increase the difficulty of treatment. In order to reduce the rate of misdiagnosis and missed diagnosis and to better help patients, we analyzed the factors affecting the risk of PE.

D-dimer is a biomarker of fibrin formation and degradation (Halaby et al., 2015) and is highly sensitive to thrombosis. It has a high sensitivity (approximately 96%) but a low specificity (approximately 40%) in the diagnosis of PE (Glober et al., 2018; Lei et al., 2021). Pregnancy, cancer, venerable age, chronic inflammatory conditions, and many other illnesses can also increase the content of D-dimer (Choi and Krishnamoorthy, 2018; Aranda et al., 2021), which brings more difficulties to diagnosing PE. According to a retrospective study in 2019, a low pretest clinical probability score (traditional Wells) in a patient with a D-dimer level of less than 1,000 ng per milliliter and a moderate pretest clinical likelihood score in a patient with a D-dimer level of less than 500 ng per milliliter could rule out PE and be protected from radiation (Kearon et al., 2019). A D-dimer concentration greater than 3,000 microg/ml in patients with chronic PE is highly associated with acute PE, and these patients must be hospitalized (Agterof et al., 2009). Non-ICU COVID-19 patients with a D-dimer level ≥2,000 ng/mL are considered to need further examination to rule out PE (Thoreau et al., 2021). Data from a 3,619 study of PE showed that high D-dimer levels had almost been proved to be significantly associated with the severity of PE (Hou et al., 2021). Another trial looked at the percentage decrease in D-dimer concentration between at diagnosis and within 1 month of diagnosis. After a month of anticoagulant therapy, D-dimer decreased by 76.6% for complete recanalization and 31.4% for residual thrombosis. Pulmonary artery recalculation was achieved in 73 of 80 patients (91.2%) with a 70% or more decrease in D-dimer concentration. Measuring the degree of D-dimer decline can aid clinicians forecast the risk of recurrent PE (Aranda et al., 2021). Several more studies have shown that there was a correlation between D-dimer level and short-term or 3-month mortality (Klok et al., 2008a; Agterof et al., 2009; Kline, 2009; Singanayagam et al., 2011; Bi et al., 2021). Elevated plasma D-dimer level was positively associated with 3-month mortality in APE (Becattini et al., 2012). A variety of factors affecting a D-dimer level make us more cautious in clinical encounters with patients with elevated D-dimer concentration.

Albumin, which is the most abundant circulating protein in the body and occupies about 50% of the whole protein content in plasma, plays a critical role in the physiological stage of the body, including osmotic effect, anti-inflammatory activity, anti-thrombus formation, antioxidation and carriers of endogenous and exogenous substances (Caraceni et al., 2013; Arroyo et al., 2014; Hoskin et al., 2020). The first study to find that hypoproteinemia is an independent predictor of long-term mortality from acute pulmonary embolism was conducted in 2018 (Tanik et al., 2020). In addition, this also applies to patients with postoperative acute pulmonary embolism (Pan, 2012). As indicated by the laboratory metrics in Table 3, albumin concentrations in the high-risk group were lower than those in the intermediate-low-risk group (*P*-values < 0.05). The results were the same with previous studies. For every 1 gm/dL reduction in albumin concentration, the possibility of a massive APE was 75% more likely (Omar et al., 2020), while with each unit increase in albumin concentration, the probability of death decreased by approximately 15.4% (Liu et al., 2020). Pulmonary embolism can cause inflammation of pulmonary blood vessels and parenchyma (Celik et al., 2021). Until now, a large amount of evidence makes clear that inflammatory response has been considered closely related to VTE. Inflammatory markers, involving C-reactive protein (CRP), tumor necrosis factor alpha, interlenkin-6 (IL-6), and interlenkin-8 (IL-8), be capable of triggering the coagulation system by inducing the expression of tissue factors (Branchford and Carpenter, 2018). Furthermore, elevated white blood cell count is strongly associated with recurrent VTE in cancer patients (Trujillo-Santos et al., 2008) and can lead to poor prognoses in patients with PE (Sanchez et al., 2008). Apparent higher CRP and white blood cell counts in patients with lower serum albumin levels may be one of the reasons for the higher mortality (Kennedy and Eberhart, 1995). Reduced anti-inflammatory capacity in patients with hypoproteinemia also increases the severity of PE (Hoskin et al., 2020). Serum albumin performs antioxidant functions by providing 80% of extracellular thiols, providing sulfur-containing amino acids for glutathione, binding and inactivating free metals (copper and iron etc.) and capturing free radicals (Bruschi et al., 2013; Taverna et al., 2013; Arroyo et al., 2014). Elevated reactive oxygen species and myeloperoxidase levels can cause lung damage (Ovechkin et al., 2007). So, we can speculate that reduced antioxidant capacity in patients with hypoproteinemia may be another reason for the poor prognosis in patients with APE. On the one hand, serum albumin plays a role in dilating blood vessels and inhibiting platelet aggregation by acting as a storage, carrier, and supplier role for nitric oxide (NO) and eicosanoids (Arroyo et al., 2014). On the other hand, the inhibitory function of albumin on platelet aggregation were related with reducing the production of thromboxane A2 and promoting endoperoxides to prostaglandin D2 transformation. More importantly, plasma albumin is inhibitable to platelet-activating factor (Mikhailidis and Ganotakis, 1996). This may be another explanation for the association of low albumin levels with poor prognosis.

History of the tumor as a risk factor for poor prognosis in sick people with PE has been reported in considerable literature. In the autopsy of 127,945 cancer patients, 5.3% of the deaths were related to PE (Valerio et al., 2021). Pancreatic cancer, brain cancer, and multiple myeloma have the highest risk of VTE (Klok et al., 2008b), especially in the first year of diagnosis (Geerts et al., 2008; Cronin-Fenton et al., 2010), whereas hematologic and breast malignancies have an lower risk (Shinagare et al., 2011). Among the pathology of lung cancer, adenocarcinoma is the most associated with PE (Cui et al., 2021). This may be caused by adenocarcinoma cells secreting mucin, activating blood platelets, and the thrombogenic mediator (Geerts et al., 2008). In addition, D-dimer, stages III–IV, DVT, low arterial partial oxygen pressure (PaO_2_), chemotherapy, and hyperleukocytosis are also considered risk elements for PE in lung cancer sick people (Chuang and Yu, 2009; Li G. et al., 2017; Ma and Wen, 2017; Cui et al., 2021). Data from a previous study showed that the median survival time of the PE group (*n* = 30) was remarkably shorter than that of the non-PE group (*n* = 60, *P* < 0.05) (Ma and Wen, 2017). Another study also confirmed this (Li G. et al., 2017). Compared to patients without cancer, patients with cancer showed less severe PE, which may also be one of the reasons for the poor prognosis due to the untimely detection of PE (Au et al., 2021). Cancer complicated with PE increases treatment difficulty and shortens the survival time. If cancer patients have unexplained dyspnea and increased D-dimer (>2.0 g/ml) (Kwon et al., 2021), it is time to perfect CTPA as possible. Early detection, diagnosis, and timely anticoagulation symptomatic treatment can remarkably improve the prognosis and prolong the survival time of cancer patients.

If the thromboembolic area exceeds 30–50% of the pulmonary artery bed (Keller et al., 2015), mechanical factors, neurohumoral factors, and hypoxia contribute to pulmonary artery contraction, resulting in increased pulmonary vascular resistance (Smulders, 2000; Lankhaar et al., 2006) and subsequently increased pulmonary artery pressure. After that, the increase of right ventricular afterload and ventricular wall tension leads to right ventricular enlargement, resulting in ventricular septum shift and following impaired left ventricular function. As a result, cardiac output is reduced, which is the cause of systemic hypotension or shock when a pulmonary embolism occurs (Mebazaa et al., 2004; Chin et al., 2005; Gok et al., 2020; Konstantinides et al., 2020). Right ventricular dysfunction (RVD) and hypotensive shock are the main cause of death from APE, which has been shown in many previous studies. Based on data from a study of 39,257 APE patients, 30-day PE-related mortality in patients with systolic SBP < 90 mmHg was 2.6 times higher than in other patients (absolute risk, 13.6%). Moreover, they also found that patients with an SBP <70 mmHg had the highest mortality while those with an SBP ≥130 mmHg had a lower mortality rates (Quezada et al., 2020). Ates et al. (2017) found that blood pressure index (BPI), with the <1.4 cut-off level, can be used as a predictor of mortality from APE, with a sensitivity of 60.6% and specificity of 80.8%. High central venous pressure and low cardiac output secondary to (RVD) can lead to reduced renal blood flow and, thus, enhanced blood urea nitrogen (BUN) (Gok et al., 2020). There has been evidence that increased BUN is highly related to APE mortality (Tatlisu et al., 2017; Gok et al., 2020; Fang and Xu, 2021), and BUN was 34.5 mg/dL as the optimal cutoff value for predicting in-hospital mortality for APE (Tatlisu et al., 2017). To reduce mortality, PE patients with hemodynamically unstable need further assessment to determine whether there are indications for thrombolytic therapy in addition to general supportive and anticoagulant therapy (Schellhaass et al., 2010).

The clinical symptoms and signs of PE lack specificity and often manifest as dyspnea, chest pain, hemoptysis or syncope. Several clinical studies have shown that the most common clinical symptom in patients with PE is dyspnea, followed by chest pain, while syncope (4–17%) and hemoptysis are relatively rare (Pollack et al., 2011; Pribish et al., 2020; Richmond et al., 2021). Syncope in PE patients may be caused by cerebral hypoperfusion due to reduced cardiac output, arrhythmias due to hemodynamic instability, or neurogenic syncope (Keller et al., 2018; Siddappa Malleshappa et al., 2020). Inadequate oxygen supply due to hypoxemia is also a cause of syncope (Keller et al., 2018; Pop et al., 2019). In the emergency department, 2.2% of syncope patients were eventually diagnosed with PE (deSouza, 2020). Furthermore, another study was 1.4% (Badertscher et al., 2019). de Winter et al. (2020) found in a meta-regression analysis that patients with pulmonary embolism associated with syncope had higher short-term mortality, which was associated with hemodynamic instability. Another study found that syncope’s prediction of 30-day mortality from pulmonary embolism was gender-specific, holding true in women but not in men. In addition, laboratory tests in patients with syncope showed higher levels of D-dimer and troponin T, and RVD was more pronounced in women with syncope. Dzudovic et al. (2020) also proposed that the increased mortality of high-risk PE patients with syncope was caused by RVD and hemodynamic instability, while the mortality of sick people with low and medium risk of PE was not significantly increased (Mohebali et al., 2020). There was no significant association between syncope and prognosis or mortality in patients with hemodynamically stable pulmonary embolism (Barco et al., 2018). These may be related to the pathophysiological mechanism of syncope. Patients with PE who take syncope as the first symptom are at higher risk of cerebral hemorrhage, so neurological examination should be emphasized in these patients (Chopard et al., 2021).

## 6 Conclusion and future works

The paper proposes the SSACS-KELM classification prediction model with high accuracy to effectively select the key features in the PE dataset to assist physicians in diagnosing pulmonary embolism. First, this paper details the actual sampling method and sampling locations of the PE dataset to ensure the authenticity and validity of the original data. Then, to enhance the accuracy and stability of the traditional feature selection approach, this paper proposes optimizing the traditional KELM classifier by using the swarm intelligence method. Further, to address the shortcomings of the swarm intelligence algorithm, an enhanced variant of SSACS is presented. In the experimental part, the optimization performance of SSACS is first verified by 30 benchmark functions and 10 peer swarm intelligence algorithms. Then, the adaptability and accuracy of the SSACS-KELM model for different datasets are verified using eight public datasets. Then, this paper shows the superiority of SSACS-KELM through comparison experiments with five traditional feature selection methods, five machine learning classifiers, and nine KELMs based on swarm intelligence algorithms. Finally, the key features, Syncope, HOT, SBP, D-D, and ALB, are finally derived by a 10-fold cross-validation method. A careful discussion proves that these five features are also fully compatible with medical facts and statistical results. In conclusion, the benchmark function performance of SSACS is very strong, and the classification accuracy of SSACS-KELM is very excellent, which is expected to be an effective model for PE diagnosis.

The proposed method also has some limitations. First, the combination of the CS algorithm and the SSA increases the complexity level of the original algorithm. This problem can be solved by parallel computing or with the rapid exploitation of computer technology and the continuous improvement of computer computing power. Second, the optimization performance of the SSACS algorithm has been proven in the medical field, and the others are only theoretical evaluations. In future work, SSACS will be applied to image segmentation, engineering optimization, and other problems. Finally, we will delve into classification and prediction in machine learning based on SSACS for other fields, such as new energy and agriculture.

## Data availability statement

The original contributions presented in this study are included in the article/supplementary material, further inquiries can be directed to the corresponding authors.

## Ethics statement

The Ethics Committee of the Affiliated of the Wenzhou Medical University agreed the present study (ethical approval code: KY2021-R097).

## Author contributions

HS, ZH, YF, AH, PW, and YC: writing – original draft, writing – review and editing, software, visualization, and investigation. DZ, YS, and HC: conceptualization, methodology, formal analysis, investigation, writing – review and editing, funding acquisition, and supervision. All authors contributed to the article and approved the submitted version.
